# Flies use acetic acid to protect their offspring from parasitoids

**DOI:** 10.1371/journal.ppat.1013368

**Published:** 2025-08-11

**Authors:** Kayla F. Reddy, Aleksey Prok, Corinne M. Stouthamer, Todd A. Schlenke

**Affiliations:** 1 Department of Entomology, The University of Arizona, Tucson, Arizona, United States of America; 2 Department of Entomology, The University of Georgia, Athens, Georgia, United States of America; University of Kansas, UNITED STATES OF AMERICA

## Abstract

Plants and fungi often produce toxic metabolites, but herbivores and fungivores that evolve resistance to these toxins gain access to underutilized resources. An additional benefit of living in and consuming toxins is that animals can gain protection against non-resistant predators and parasites. The fruit fly *Drosophila melanogaster* consumes yeasts growing on rotting fruit and has evolved resistance to toxic fermentation products such as ethanol and acetic acid. We tested whether acetic acid protects flies from one of their most common natural enemies, parasitoid wasps, which infect fly larvae and pupae. We found that both wasp parasitism rate and wasp eclosion success are reduced when fly larvae are grown on acetic acid food, and wasp mothers actively avoid infecting fly larvae reared in acetic acid food if given a choice. In each case, acetic acid results in a greater fitness cost for a generalist parasitoid compared to a specialist parasitoid. Furthermore, fly mothers sense the presence of parasitoids in their environment and alter their oviposition behavior to lay eggs in more acetic acid-heavy food when wasps were present. This demonstrates that flies perceive the competing costs to their offspring of wasp parasitism and acetic acid toxicity but balance those costs to maximize offspring fitness.

## Author summary

When organisms evolve resistance to the toxic metabolites of plants and fungi found in their environment, they gain access to new resources and also gain protection from non-resistant predators and parasites. The fruit fly *Drosophila melanogaster* lives in and consumes rotting fruit, and has evolved resistance to fermentation products such as ethanol and acetic acid. We tested whether acetic acid protects flies from one of their most common enemies: parasitoid wasps. We found that acetic acid is toxic to juvenile wasps living inside their fly hosts, and that adult wasps avoid infecting flies that are in food containing high levels of acetic acid. These toxic effects of acetic acid were more pronounced for a generalist parasitoid of *D. melanogaster* compared to a specialist parasitoid. Furthermore, adult flies prefer to oviposit in food containing higher levels of acetic acid when wasps are present, suggestions that they balance the fitness costs of acetic acid toxicity versus wasp parasitism when deciding where to place their offspring.

## Introduction

Parasitoid wasps are exceedingly diverse and infect most insect species [1–3]. The fruit fly *D. melanogaster* is host to parasitoids from at least four wasp families [4], and upwards of 50% of fly offspring may be infected by wasps in natural settings [5–7]. When parasitoids inject an egg into a fly larva, the egg hatches and the wasp larva begins consuming fly hemolymph, allowing the fly to continue collecting food as usual. Once the fly pupates, however, the wasp larva begins consuming fly hard tissues, killing the host and eventually eclosing as an adult wasp from the fly pupal case. Because wasp parasitism results in fly death, flies experience strong selection pressures to protect themselves and their offspring from parasitism.

Flies mount an immune response against parasitoid eggs termed ‘encapsulation’, whereby thousands of hemocytes (blood cells) migrate towards, adhere to, and consolidate in a capsule around the wasp egg. The hemocytes then release melanin and free radicals onto the wasp egg to asphyxiate, damage, and physically prevent it from hatching [8]. This immune response is energetically costly and not guaranteed to succeed since wasp mothers inject venom into hosts along with their eggs to suppress encapsulation [9–15]. However, flies have also evolved inducible behaviors that allow them to prevent parasitism or potentially cure themselves once infected. For example, fly larvae avoid wasp odors and mount a rolling behavior when pierced by parasitoid ovipositors to try to dislodge the infecting wasp [16,17]. Likewise, fly mothers avoid laying eggs at sites containing wasp odors and lay fewer eggs in the forced presence of wasps [16,18].

Toxic metabolites have been shown to harm endoparasitoids in other host-parasitoid systems [19–22]. Adults of the fruit fly *D. melanogaster* consume yeasts growing on rotting fruit, they lay their eggs on rotting fruit, and once hatched the fly offspring complete their larval development in the rotting fruit. Fermentation, or the production of ATP via the anaerobic degradation of organic nutrients, is a common form of energy generation used by bacteria and yeasts in these rotting fruit environments. Thus, *D. melanogaster* has evolved a remarkably high tolerance of ethanol and acetic acid, two common byproducts of fermentation [23–26]. We previously discovered that flies use this ethanol tolerance to their advantage to fight parasitism: Fly larvae actively seek out and consume alcohol once infected by parasitoid wasps, as the buildup of alcohol in their hemolymph kills developing wasp larvae [27]. Furthermore, high alcohol environments protect fly larvae from getting infected in the first place, and fly mothers preferentially lay their eggs in habitats containing higher concentrations of alcohol when parasitoids are present in their environment [28,29], which the flies sense using both sight and smell [16,28]. These behavioral adaptations in *D. melanogaster* are more successful against generalist parasitoids than specialist parasitoids, likely because specialist parasitoids have also been heavily selected to evolve tolerance of fermentation byproducts [30–32].

Acetic acid levels found in natural *D. melanogaster* habitats range up to 5% by volume in rotting fruits and may be substantially higher in microenvironments (e.g. tunnels) within the rotting fruits [25]. Flies are attracted by the smell of acetic acid as an indication that they are nearing an attractive rotting fruit [33–38], and fly consumption of food with moderate levels of acetic acid can result in increased fitness, as is the case with ethanol [37,39,40]. However, consumption of higher acetic acid concentrations (i.e., greater than 5%) causes increasing fly mortality [25,36,37].

Given that alcohol can protect flies from wasp parasitism, we decided to test whether acetic acid also provides protection. We used two closely related parasitoid wasp species: *Leptopilina boulardi* is a specialist parasite of *D. melanogaster* and its close relatives whereas *L. heterotoma* is a generalist parasite that infects a diversity of *Drosophila* species living in fermenting fruits, decaying plant materials, and sap fluxes [4,30,31,41]. Both wasp species are attracted to the odor of fermentation products such as acetic acid, presumably as a means to locate hosts, and they are each highly infectious in *D. melanogaster* lab strains [41–44]. We find that fly larvae, once infected, are not protected by consumption of acetic acid food. However, acetic acid environments protect fly larvae from becoming infected by parasitoid wasps, and when fly mothers sense wasp presence they begin laying eggs in food with higher acetic acid concentrations.

## Results

### Acetic acid reduces parasitoid wasp developmental success

**Fig 1:**
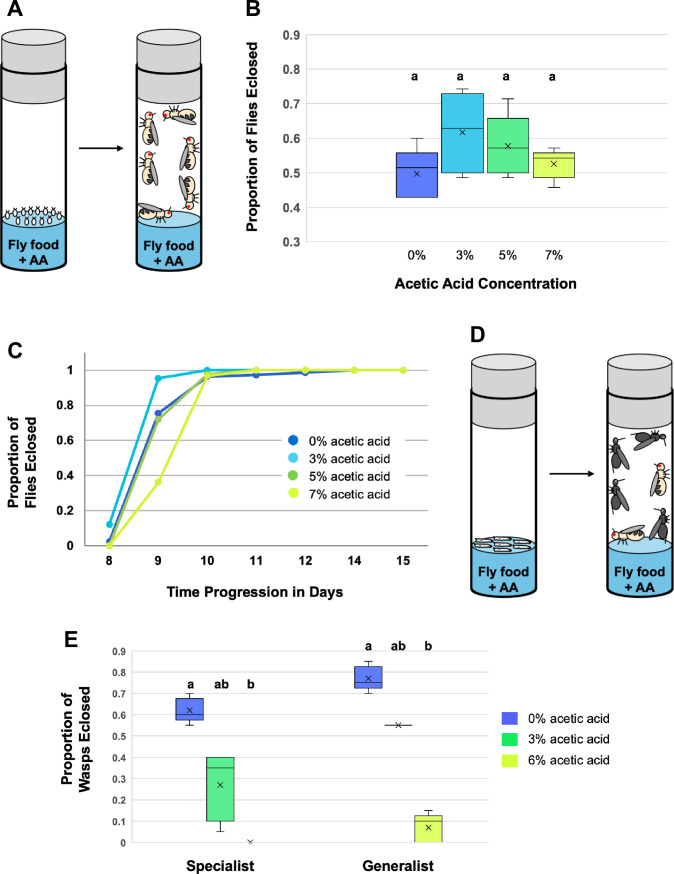
Acetic acid (‘AA’) effects on insect development. (A) Cartoon demonstrating measurement of fly egg-to-adult eclosion success in food containing different concentrations of acetic acid. (B) Box and whisker plot showing proportion of fly eggs that survived into the adult stage depending on acetic acid concentration. The median, quartiles, and outlier ranges are shown. Dunn’s test was used to identify significance groupings. N = 5 replicates for each treatment. (C) Based on the same experiment as above, fly development to the eclosion stage was measured over time based on food acetic acid concentration. (D) Cartoon demonstrating measurement of wasp egg-to-adult eclosion success in food containing different concentrations of acetic acid. The first vial contains fly larvae infected by wasp eggs. (E) Box and whisker plot showing proportion of wasp eggs (living inside flies) that survived into the adult stage depending on acetic acid concentration. The median, quartiles, and outlier ranges are shown. Dunn’s test was used to identify significance groupings within each wasp dataset. N = 5 replicates for each treatment.

To determine the effect of different acetic acid concentrations on fly larval development, we placed groups of fly eggs in Drosophila vials containing food with varying acetic acid concentrations, then measured fly eclosion over time (Fig 1A). There was no significant difference in fly eclosion success with the concentrations of acetic acid that we tested (Kruskal-Wallis test, H = 3.69, p = 0.30) (Fig 1B). Fly eclosion success was lowest in the control (0% acetic acid) treatment, but this was likely because of the growth of contaminating fungus in the fly media not containing any acetic acid. Of the flies that survived to eclosion however, there were significant differences in fly eclosion timing across different acetic acid concentrations (Fig 1C). Flies exposed to 3% acetic acid eclosed quickest, flies exposed to 0% or 5% acetic acid eclosed at similar intermediate rates, and flies exposed to 7% acetic acid eclosed the slowest, approximately one day after flies from the 3% acetic acid treatment (Kruskal-Wallis test, H = 14.26, p = 0.003 for day 9, all other days non-significant). These data suggest that fly larvae can tolerate relatively high levels of acetic acid, though it does slow their development. But what about wasps living inside fly larvae?

Wasp eggs laid inside fly larvae hatch and grow within the hemocoel of infected fly larvae for several days. At this point, older fly larvae enter the ‘wandering phase’ and move out of the food to pupate. To determine whether juvenile wasps suffer increased mortality when their fly hosts live in acetic acid-laden food, we infected fly larvae in acetic acid-free food, then moved the infected fly larvae onto new food with varying concentrations of acetic acid and allowed them to complete development (Fig 1D). Over 70% of the infected flies pupated in all treatment replicates. However, as acetic acid level increased, the number of eclosing adult wasps significantly decreased for both the specialist wasp *L. boulardi* and the generalist wasp *L. heterotoma* (specialist: Kruskal-Wallis test, H = 12.5, p = 0.002; generalist: Kruskal-Wallis test, H = 12.5, p = 0.002) (Fig 1E). These data indicate that flies that consume acetic acid make poorer hosts for the parasitoids, perhaps because the acetic acid contributes to a toxic environment inside the fly bodies where the parasitoids are developing. However, the number of infected flies that successfully eclosed did not increase with increasing acetic acid levels (fly eclosion success was less than 15% for all treatment replicates), suggesting that the wasps lived long enough (through fly pupation) to cause irreparable harm to their hosts before they die. This finding argues against the possibility that fly larvae might use acetic acid to medicate themselves once infected.

### Acetic acid protects fly larvae from wasp parasitism

**Fig 2:**
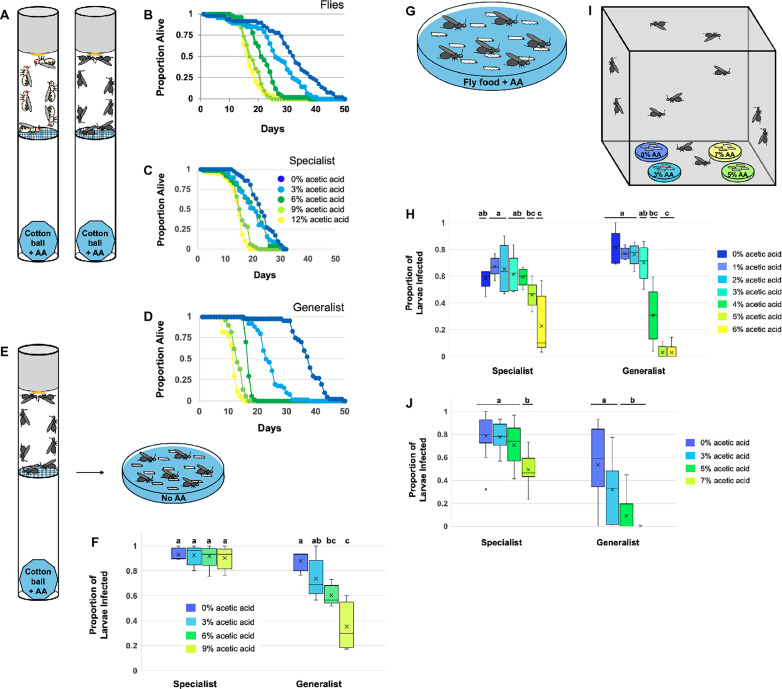
Effects of acetic acid on adult insects. (A) Cartoon demonstrating our method of assaying acetic acid vapor toxicity on flies and wasps. (B-D) Survival curves for flies and wasps exposed to different concentrations of acetic acid vapor. The acetic acid concentration key is found in (C). N = 5 replicates for each treatment. (E) Cartoon demonstrating how we determined whether exposure to acetic acid vapor affected wasp ability to infect flies. (F) Box and whisker plot showing wasp success at infecting flies after acetic acid vapor exposure. The median, quartiles, and outlier ranges are shown. Dunn’s test was used to identify significance groupings. N = 5 replicates for each treatment. (G) Cartoon demonstrating wasp parasitism trials with fly larvae living on varied acetic acid concentrations. (H) Box and whisker plot showing wasp success at infecting flies depending on the acetic acid concentration the fly larvae are living in. The median, quartiles, and outlier ranges are shown. Dunn’s test was used to identify significance groupings within each wasp dataset. N = 5 replicates for each treatment. (I) Cartoon demonstrating wasp oviposition choice trials where wasps can choose whether to infect fly larvae in food containing different acetic acid concentrations. (J) Box and whisker plot showing proportion of fly larvae in food containing different acetic acid concentrations that were infected by wasps. The median, quartiles, and outlier ranges are shown. Dunn’s test was used to identify significance groupings within each wasp dataset. N = 16 replicate cages per wasp species.

The results above demonstrate that although being in acetic acid-laden food does not increase larval fly survival once infected, it does hurt wasp survival, which may select for adult wasps to avoid infecting fly larvae when they are living in acetic acid-laden food. Alternatively, acetic acid may be toxic enough to adult wasps that they are unable to infect fly larvae living in food with high acetic acid concentrations. To gain insight into acetic acid toxicity in adult insects, we exposed insects to different concentrations of acetic acid vapor. Insects were placed in vials separated by mesh from an acetic acid-soaked cotton ball placed at the base of a connected vial (Fig 2A). This ensures that the flies and wasps are exposed to acetic acid vapor without the possibility of coming into physical contact with acetic acid. Surviving insects were counted daily until all insects had perished. Survival curves show that all three insect species (fly, specialist wasp, generalist wasp) suffered decreased survival as acetic acid concentrations increased (Fig 2B-2D). The generalist wasp *L. heterotoma* survived the longest of the three insects when in acetic acid-less control vials with an average lifespan of 37 days. However, it also showed the greatest reduction in survival due to increasing acetic acid levels, with an average lifespan of only 12 days at the highest acetic acid concentration compared to 17 days for *D. melanogaster* and 15 days for the specialist wasp *L. boulardi*. Although acetic acid reduced insect lifespan, almost all insects survived a week of exposure to even the highest concentration of acetic acid vapor, indicating that vapor exposure is not acutely toxic.

Nevertheless, we questioned whether long-term acetic acid vapor exposure would harm adult wasp ability to infect fly larvae. We exposed adult wasps to acetic acid vapor as above for three days. The wasps were given an hour to recover, then allowed to infect fly larvae in an acetic-acid free environment (Fig 2E). Fly larvae were then dissected and scored for wasp eggs to determine parasitism rate. The ability of the specialist wasp *L. boulardi* to parasitize fly larvae remained unchanged (Kruskal-Wallis test, H = 0.4, p = 0.94), but the ability of the generalist wasp *L. heterotoma* to parasitize decreased as acetic acid level increased (Kruskal-Wallis test, H = 13.7, p = 0.003) (Fig 2F). This indicates that, at least for the generalist wasp, chronic exposure to acetic acid reduces their parasitism capacity.

We next tested how well naïve wasps were able to infect fly larvae living in food with different acetic acid concentrations. In this scenario, wasps must cope with an acute exposure to both acetic acid vapor and contact toxicity to their tarsi and ovipositor. We placed fly larvae in petri dishes containing media with different acetic acid concentrations, exposed the fly larvae to wasps, then dissected open the flies to determine whether they were infected with wasp eggs (Fig 2G). For both wasp species, the parasitism rate significantly decreased as the acetic acid concentration increased (specialist: Kruskal-Wallis test, H = 17.4, p = 0.008; generalist: Kruskal-Wallis test, H = 27.0, p = 0.0001) (Fig 2H). However, while there was no clear dropoff in parasitism rate of the specialist wasp *L. boulardi* until the 6% acetic acid level, the generalist wasp *L. heterotoma* showed a significant decrease in parasitism rate at the 4% acetic acid level, indicating that the generalist wasp was less able to maintain its parasitism ability in the acetic acid-containing food than the specialist wasp.

Given that wasps are less successful at parasitizing host fly larvae when the larvae are living in acetic acid-laden food, we next asked whether if, given a choice, the wasps prefer to infect hosts living in food with lower levels of acetic acid. Fly larvae were placed in dishes containing acetic acid at four different concentrations and these sets of four dishes were placed inside mesh insect cages. Wasps were then introduced to the cages and given the opportunity to parasitize, with equal access to each of the four different dishes (Fig 2I). After the parasitism period the fly larvae were frozen and later dissected to record the number of wasp eggs. There were significant differences in the proportion of fly larvae that got infected in the different acetic acid concentration dishes for both wasp species (specialist: Kruskal-Wallis test, H = 24.2, p = 0.00002; generalist: Kruskal-Wallis test, H = 30.5, p = 0.000001) (Fig 2J). The specialist wasp infected a large proportion of the fly larvae at all acetic acid concentrations, although the proportion of infected flies dipped below 50% at the highest acetic acid level (7%). However, the generalist wasp *L. heterotoma* showed a lower parasitism rate overall and was much less likely to infect flies as food acetic acid concentration increased, with zero evidence of parasitism in flies at the highest acetic acid level (7%). These results indicate that wasps, especially the generalist wasp, sense localized differences in acetic acid concentration and make choices in real time about which hosts to infect based on acetic acid level.

### Flies choose more toxic oviposition sites when parasitoid wasps are present

**Fig 3:**
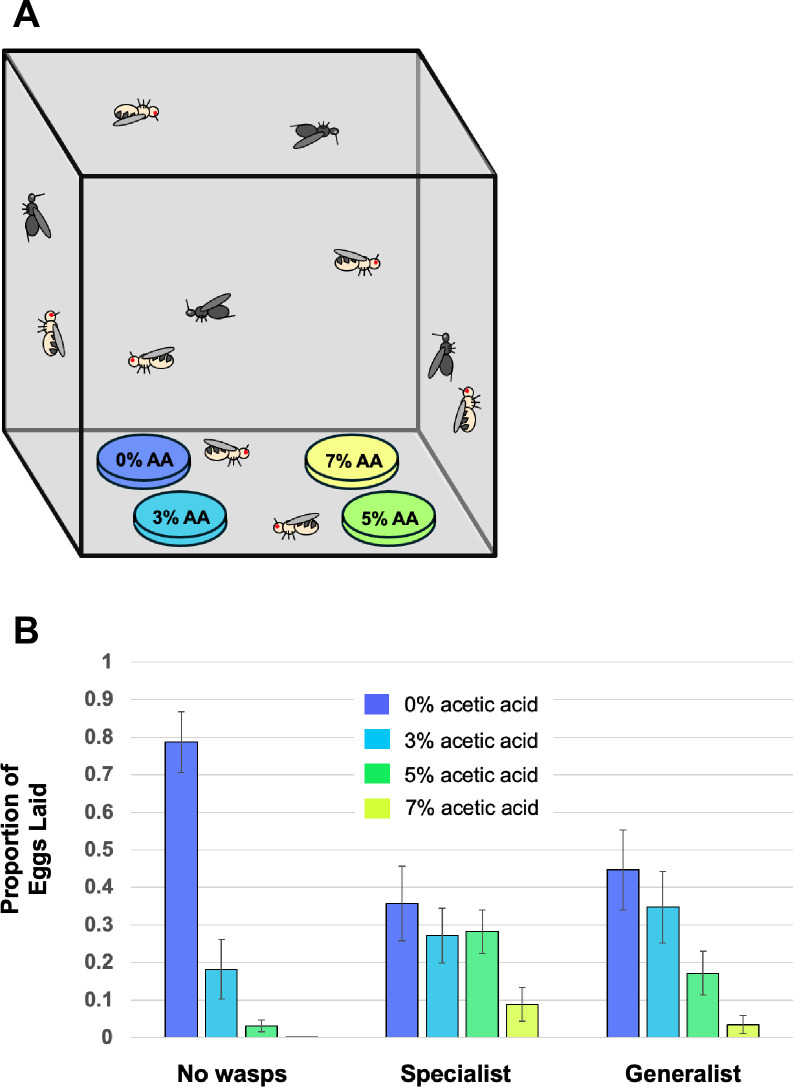
Fly choice of oviposition sites in the presence of wasps. (A) Cartoon demonstrating the cage experiment with dishes containing different concentrations of acetic acid. (B) Proportion of eggs that flies laid in the different acetic acid concentrations based on wasp treatment. Error bars represent standard errors. N = 12 replicate cages per wasp species.

Given the protection from parasitism that acetic acid-laden food offers, we wondered whether adult flies would sense the wasps and alter their oviposition preference towards more toxic food when wasps were present. Adult flies were housed with adult wasps in mesh cages and given a choice of four oviposition sites – petri dishes containing different concentrations of acetic acid (Fig 3A). After 19 hours the number of fly eggs on each dish was recorded. When no wasps were present, flies laid the great majority of their eggs in the dish with no acetic acid (Fig 3B). When either species of wasp was present however, a significantly greater proportion of eggs were laid in the food containing acetic acid (chi square test of egg numbers in different dishes, p < 10^-5^). Regardless of wasp treatment, flies always laid the fewest eggs on the dish with the highest concentration of acetic acid. These data indicate that flies innately balance the threat to their offspring of acetic acid toxicity and parasitism, and make appropriate oviposition decisions depending on the local conditions.

## Discussion

*D. melanogaster* utilizes rotting fruit as a food source and oviposition site and has evolved tolerance of toxins in this niche, notably the toxic by-products of fermentation such as ethanol and acetic acid [25]. Evolving tolerance to environmental toxins can protect organisms from generalist predators and parasites that are not themselves resistant. In this study, we find that acetic acid is toxic to parasitoid wasps that infect *D. melanogaster*, especially to a more generalist parasitoid. Fly larvae living in food containing acetic acid are infected less. Furthermore, when adult flies perceive parasitoid wasps in their environment they undergo an adaptive behavioral shift to oviposit in foods with higher acetic acid concentrations, a form of parental care. This behavior is induced, not constitutive, presumably because of the developmental delay experienced by fly larvae living in acetic acid-laden food. Thus, the flies have evolved to balance risk of toxicity with risk of parasitism. The results further suggest that wasp populations may be patchy in space and time and that a current absence of wasps at an oviposition site is a potential indicator that wasps will continue to be absent during the window in fly larval development when they are most susceptible to wasp parasitism (second instar, approximately 2–3 days after oviposition) [4].

These results are broadly comparable to our previous work showing that ethanol protects flies from wasp parasitism. Like with acetic acid, fly larvae living in ethanol-laden food are infected less, and adult flies preferentially oviposit in ethanol-laden food when wasps are present [28]. Unlike with acetic acid, ethanol also protects fly larvae after they are infected because ethanol-induced wasp mortality enhances fly survival [27]. Although we found that acetic acid reduces wasp survival in fly larvae, the acetic acid-mediated toxicity to juvenile wasps did not enhance fly survival, perhaps because the negative effect of acetic acid on wasp development is slower to manifest than with ethanol. Given that acetic acid-laden food did not rescue infected flies in our experiments, we did not directly test whether fly larvae ‘medicate’ themselves by choosing higher concentration acetic acid food when they were infected, as was the case with ethanol.

One question that remains is how the presence of acetic acid in the fly food reduces the wasp parasitism rate of fly larvae. Acetic acid vapor does not seem to be acutely toxic to wasps, suggesting that wasps should be able to recover from short parasitism bouts by leaving the toxic area. We have not tested whether wasps have a touch sensitivity to acetic acid, and if touching the acetic acid-laden media with their tarsi is strongly aversive to the wasps. Outside of a direct toxicity to the adult wasps, the fact that wasp larvae have lower survival living inside fly larvae growing on acetic acid-laden food suggests that there is selection on adult wasps to avoid laying their eggs in hosts living in those environments. In this scenario, adult wasps would sense acetic acid in the food via olfaction of acetic acid vapor, by gustation of acetic acid through their tarsi, or by chemical receptors in their ovipositors sensing the composition of their hosts’ hemolymph. Acetic acid sensing would then cause them to seek out alternative oviposition sites. It will be interesting to tease apart these potential mechanisms for wasp oviposition decisions in future experiments.

One difficulty inherent to our lab experiments supplementing fly media with acetic acid is that acetic acid is a volatile and its level will diminish over the course of our experiments. This isn’t necessarily the case in natural settings given that fermenting bacteria living on rotting fruits will continue to generate acetic acid as a byproduct of their metabolism. Although we tried to use naturally relevant acetic acid concentrations in our experiments [25], the levels of acetic acid toxicity we observed in our experiments are likely underestimates compared to what would be observed in a natural setting where acetic acid levels are replenished by fermenting microbes. It is possible that wasp-infected flies living in acetic acid-laden food might have enjoyed a survival advantage if they were consuming a consistently high (rather than diminishing) concentration of acetic acid, as would likely be the case in a natural setting.

Overall, our work shows that adult flies have an inducible parental care behavior initiated by the presence of parasitoid wasps, whereby they preferentially oviposit in media with greater acetic acid toxicity. This adds to a growing a list of examples in which organisms adapted to tolerate harmful plant and fungal metabolites not only benefit from gaining access to an underexploited food resource, but also evolve novel anti-predator/parasite strategies based around their toxin tolerance [27,28,45,46].

## Methods

### Insects

The *Drosophila melanogaster* strain used in all experiments is an Oregon R strain that was cured of Wolbachia via addition of tetracycline to the food. It has been maintained in our lab on standard media for several years. The parasitoid wasps *Leptopilina boulardi* (strain Lb17) and *Leptopilina heterotoma* (strain Lh14) were collected by T. Schlenke in Winters, CA in 2002 [44]. *Leptopilina boulardi* is a relative specialist parasite of *D. melanogaster* and its close relatives, while L. heterotoma is a generalist that infects *D. melanogaster* and a broader range of other *Drosophila* species [4,44].

### Fly and wasp larval development in acetic acid food

The effect of acetic acid on fly development was measured by placing *D. melanogaster* eggs in 25mm diameter vials containing fly media mixed with acetic acid (Fig 1A). For each vial, we added 2g of Instant Drosophila Medium (Carolina Biological Supply 4–24) and pipetted 10mL of acetic acid-water mixture onto it to hydrate the food. The acetic acid mixture was made by first mixing a small amount (2.75%) of baker’s yeast in water (for fly nutrition), autoclaving this mixture to kill the yeast, then adding the appropriate amount of acetic acid to make 0, 3, 5, and 7% acetic acid concentrations. Propionic acid at a concentration of 0.11% was also added to prevent fungal contamination of the fly media. To generate fly eggs, adult flies were allowed to lay eggs in a 100mm diameter media-filled petri dish overnight. Groups of 35 fly eggs were then placed in each experimental vial, which were kept in a 25C incubator with a 12:12 light cycle. There were five replicates per acetic acid concentration for a total of 20 experimental vials. Fly developmental progress was assessed by counting newly eclosed flies each day.

The effect of acetic acid on wasp development was measured by infecting flies in an acetic acid-free environment, then moving the infected fly larvae to media with different acetic acid concentrations (Fig 1D). To generate infected flies, adult flies were allowed to lay eggs in a 100mm diameter media-filled petri dish overnight. Two days later, 375 size-matched second instar fly larvae were moved into a new 100mm diameter media-filled petri dish. Fifty female wasps were ‘experienced’ on the leftover fly larvae for thirty minutes before giving them access to the 375 experimental flies for two hours. To ensure that the majority of these fly larvae were infected, we dissected twenty of them and found that >90% of them contained wasp eggs. Groups of twenty infected fly larvae were placed into 25mm diameter vials containing Instant Drosophila Medium combined with acetic acid-water mixtures of various concentrations (0, 3, and 6%) as described above, and the vials were kept in a 25C incubator with a 12:12 light cycle. There were five replicates per acetic acid concentration for a total of 15 experimental vials for each wasp species. Wasp developmental success was measured by recording the number of fly pupae as well as the number of adult flies and wasps that eclosed from each vial.

### Adult fly and wasp tolerance of acetic acid vapor

Insect tolerance of acetic acid vapor was measured by placing the insects in an enclosure composed of two 25mm diameter fly vials. In the first vial, a cellulose acetate plug was pushed fully into the vial and then a drop of 50% honey water was pipetted onto it as sustenance – this is where the insects were housed. In the second vial, a 1g cotton ball was pushed fully into the vial and then 9mL of acetic acid-water mixture was pipetted into the cotton. The two vials were then taped together, separated by a nylon tulle mesh, with the insect vial on top of the acetic acid vial (Fig 2A).

Each insect vial housed a group of ten age-matched adult females. Five different acetic acid concentrations were used (0, 3, 6, 9, and 12%), and there were five replicates of each treatment for each insect species, for a total of 75 experimental enclosures. These enclosures were kept in a 25C incubator with a 12:12 light cycle. To measure acetic acid-mediated mortality, the number of live insects in each enclosure was counted each day.

### Wasp parasitism ability after long-term acetic acid vapor exposure

The long-term effect of acetic acid vapor on wasp parasitism ability was measured by exposing wasps to acetic acid vapor in the same double-vial enclosures used above using four different acetic acid concentrations (0, 3, 6, and 9%) (Fig 2E). With five replicates of each exposure treatment, there were twenty total exposure vials for each wasp species. After three days, five wasps were removed from each of these exposure vials and allowed to recuperate for one hour in empty 25mm diameter vials with a drop of 50% honey water in the cellulose acetate plugs. Then, each group of five wasps was placed into a petri dish containing thirty fly larvae in food containing no acetic acid. To produce these age-matched fly larvae, adult flies were allowed to lay eggs in a 100mm diameter media-filled petri dish overnight. Two days after egglay, the second-instar fly larvae were moved into new 35mm diameter petri dishes to be exposed to the experimental wasps for 2.5 hours in a 25C incubator. Each dish contained 0.5g Instant Drosophila Medium combined with 2.5mL of water, then an approximately 5mm width of food was removed from the outside edges of the dishes to leave a disc of food in the middle of the dish allowing the wasps easy access to the fly larvae. The dishes were then frozen at -80C to halt fly developmental progress so that they could be dissected later. To determine parasitism rate in each dish, each fly larval body was opened with fine forceps under a dissecting microscope and the presence of any wasp egg was tabulated.

### Effect of acetic acid on wasp parasitism rates

Wasp parasitism rate of flies grown on media with different acetic acid concentrations was measured by dissecting wasp-exposed flies to reveal the presence of wasp eggs (Fig 2G). To produce age-matched fly larvae, adult flies were allowed to lay eggs in a 100mm diameter media-filled petri dish overnight. Two days later, groups of thirty second-instar fly larvae were moved into new 35mm petri dishes containing media with a range of acetic acid concentrations (0, 1, 2, 3, 4, 5, and 6%). Each dish contained 0.5g Instant Drosophila Medium combined with 2.5mL of acetic acid/water mixture, then an approximately 5mm width of food was removed from the outside edges of the dishes to leave a disc of food in the middle of the dish allowing the wasps easy access to the fly larvae. Four experienced wasps were added to each dish. Four wasps of either species can easily infect thirty host larvae in a short period, but *L. boulardi* were given 1.5 hours to infect the flies whereas *L. heterotoma* were given two hours because *L. heterotoma* tends to take longer to acclimate to the vials before initiating infection [44]. Five replicate dishes of each acetic acid concentration, for a total of 35 dishes for each wasp species, were kept in a 25C incubator during the parasitism period. The dishes were then frozen at -80C to halt fly developmental progress so that they could be dissected later. To determine parasitism rate in each dish, each fly larval body was opened with fine forceps under a dissecting microscope and the presence of any wasp egg was tabulated.

### Wasp oviposition preference for flies living in food with different acetic acid concentrations

Wasp oviposition preference was tested by placing four petri dishes containing fly larvae and different concentrations of acetic acid into mesh cages, then releasing wasps into the cages and allowing them to choose which fly larvae to infect (Fig 2I). The 35mm diameter petri dishes contained 0.5g Instant Drosophila Medium combined with 2.5mL of acetic acid-water mixture with a range of acetic acid concentrations (0, 3, 5, and 7%). To produce age-matched fly larvae, adult flies were allowed to lay eggs in a 100mm diameter media-filled petri dish overnight. Two days later, groups of thirty second-instar fly larvae were moved into the dishes. The dishes were arranged in the four corners of 1ft^3^ mesh cages, and the arrangement of the different concentration dishes was purposefully shifted across replicates to negate any innate position bias in where wasps preferred to visit. Twenty experienced wasps were then placed into each cage and allowed to oviposit for 2.5 hours in a 25C incubator, after which the dishes were frozen at -80C to halt fly developmental progress so that they could be dissected later. To determine parasitism rate in each dish, each fly larval body was opened with fine forceps under a dissecting microscope and the presence of any wasp egg was tabulated. There were a total of sixteen replicate cages tested for each of the two wasp species.

### Fly oviposition choice in the presence of wasps

Fly oviposition choice was tested by placing four petri dishes containing different concentrations of acetic acid into mesh cages, then releasing adult flies and wasps into the cages and allowing the flies to choose which dishes to oviposit on (Fig 3A). The 60mm diameter petri dishes contained 2g Instant Drosophila Medium combined with 10mL of acetic acid-water mixture media with a range of acetic acid concentrations (0, 3, 5, and 7%). The dishes were arranged in the four corners of 1ft^3^ mesh cages and put in a 25C incubator with a 12:12 light cycle. The arrangement of the different concentration dishes as well as the placement of cages on different shelves was purposefully shifted across replicates to negate any innate position bias in where flies preferred to visit. Forty female and ten male adult flies were released into the cages, along with forty adult female wasps (or no wasps as a control), and the flies were allowed to oviposit on the dishes for nineteen hours. The dishes were then frozen at -80C to halt fly developmental progress so that the number of fly eggs in each dish could be counted later. There were a total of twelve replicate cages tested for each wasp treatment.

## Supporting information

S1 Data File Raw and processed data. (XLSX)
